# Does Intercommissural Distance Shortening in Bicuspid Aortic Valve Repair Improve Valve Opening Area?

**DOI:** 10.1016/j.atssr.2025.08.010

**Published:** 2025-09-01

**Authors:** Satoshi Hoshino, Satoshi Arimura, Junpei Takada, Yusei Okamoto, Shiho Mineta, Kiyotaka Iwasaki, Takashi Kunihara

**Affiliations:** 1 Department of Cardiac Surgery, The Jikei University School of Medicine, Tokyo, Japan; 2 Department of Modern Mechanical Engineering, School of Creative Science and Engineering, Waseda University, Tokyo, Japan; 3 Department of Integrative Bioscience and Biomedical Engineering, Graduate School of Advanced Science and Engineering, Waseda University, Tokyo, Japan

## Abstract

**Background:**

Standardized bicuspid aortic valve (BAV) repair makes 2 symmetrical cusps by plicating the fused cusp, which is accompanied by risk of aortic stenosis. Here, the effect of improving cusp mobility by shortening the intercommissural distance (ICD) on increasing the aortic valve area was examined in a BAV model using a pulsatile flow simulator.

**Methods:**

Six pairs of symmetrical BAV were created in a neo-Valsalva graft with bovine pericardium (free margin length, 26 mm; geometric height, 20 mm), which were incorporated into a pulsation circuit simulator. The ICD was gradually shortened, and the forward flow, leakage flow, pressure gradient, and aortic valve area were measured. The average value of a total of 18 measurements (3 for each model) was examined.

**Results:**

Forward flow remained constant and leakage increased slightly with ICD shortening, but the differences were not statistically significant (*P* = .17). Peak and mean transvalvular pressure gradient were significantly reduced by ICD shortening (peak: control 26.75 ± 4.33 mm Hg vs 22-mm ICD 23.85 ± 2.91 mm Hg, *P* < .05; mean: control 17.57 ± 3.59 mmHg vs 20-mm ICD 14.76 ± 2.40 mm Hg, *P* = .01). Aortic valve area was increased significantly by ICD shortening (control 2.03 ± 0.37 cm^2^ vs 18-mm ICD 2.71 ± 0.47 cm^2^, *P* <.01).

**Conclusions:**

With shortening of the ICD, the effect of increasing the aortic valve area and decreasing the pressure gradient was confirmed. Shortening the sinotubular junction diameter (= ICD) is important in aortic valvuloplasty for BAV.

## Visual Abstract

**Figure:**
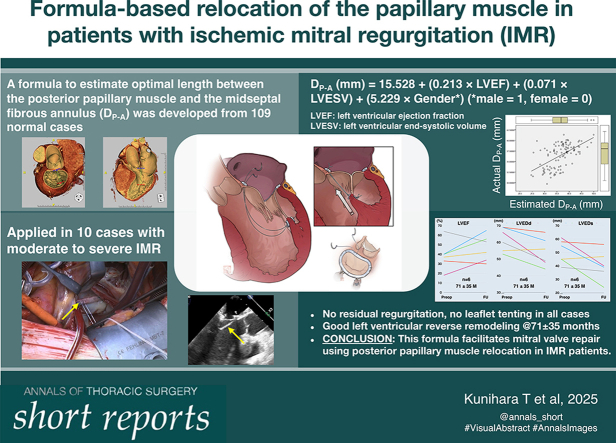

**In Short**

▪ Postoperative relevant aortic stenosis is associated with the risk of recurrence after aortic valvuloplasty for bicuspid aortic valve (BAV).
▪ Our experimental model using self-made symmetrical BAVs created in a neo-Valsalva graft with bovine pericardium that were incorporated into a pulsatile simulation circuit confirmed that shortening of the intercommissural distance significantly reduced the peak and mean pressure gradient and also significantly increased the aortic valve area.

In aortic valvuloplasty for bicuspid aortic valve (BAV), central plication is often performed on the prolapsed leaflet to achieve identical free margin length (FML) of the 2 cusps.^1^ However, the more asymmetric the cusps, the more plication stitches are required, which reduces the aortic valve area (AVA). Hence, BAVs tend to have a smaller postoperative AVA than tricuspid valves, and maximum postoperative peak transvalvular pressure gradient (PPG) >20 mm Hg after BAV repair is associated with higher risk of reoperation.^2^ A recent international consensus statement warned that creating a very asymmetric BAV with 180° symmetry increases the risk of stenosis, and therefore some countermeasures are needed.^3^

Poor valve opening occurs due to tension on the cusps, when FML is much shorter than the ideal length for intercommissural distance (ICD). We speculated that with shortening of the ICD, tension of the cusp free margin would be relaxed and the valve would open wider. However, there have been no reports supporting this hypothesis. This study was performed to examine the hydrodynamic effects of shortening the ICD in an in vitro stenotic BAV model using a pulsatile flow simulator.

## Material and Methods

This research is a basic experiment that does not involve human or animal subjects and does not require approval of the study by an institutional review board or ethics committee.

### In Vitro Stenotic BAV Model

**Figure 1:**
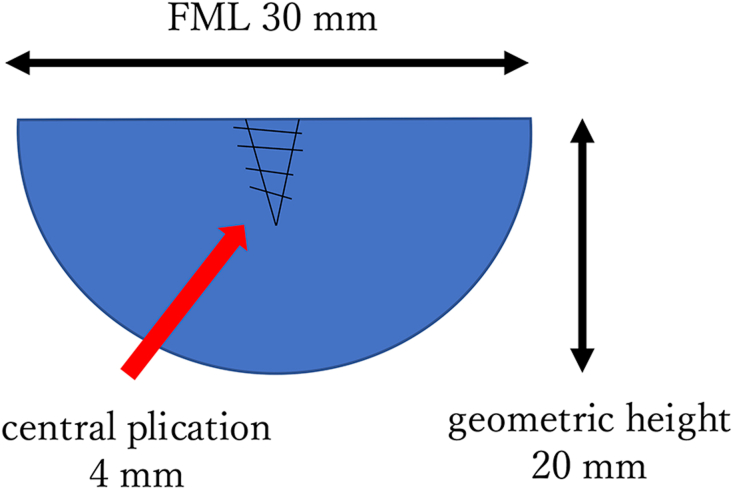
Schematic figure of a semicircular bovine pericardial patch used for the experiment. (FML, free margin length.)

**Figure 2:**
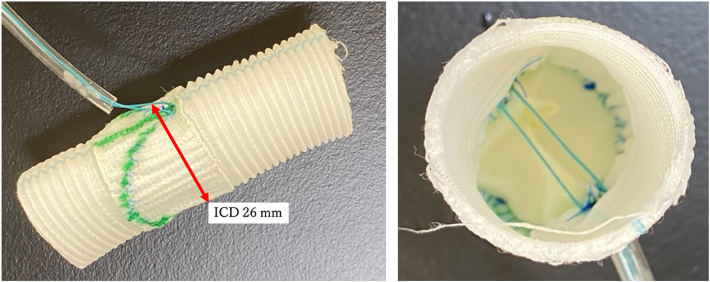
Symmetric bicuspid aortic valve model created with neo-Valsalva graft. A tourniquet was tightened between the commissures. (ICD, intercommissural distance.)

Among patients with BAV undergoing aortic valvuloplasty with symmetric bicuspidization at our hospital, the shortest FML after central plication was 30 mm, geometric height was 20 mm, and PPG of this patient was >20 mm Hg. Using this as a reference value, a pair of semicircular patches were cut from bovine pericardium (Model 4700; Edwards) (FML, 30 mm; geometric height, 20 mm) (Figure 1) and symmetrically sewn into a neo-Valsalva prosthesis (J-graft Neo Valsalva 24 mm; Japan Lifeline Co) (Figure 2). The height of the commissure was 20 mm and the diameter of the graft at this position (= ICD) was 26 mm. This in vitro BAV model was incorporated into the pulsatile simulator, and the mean transvalvular pressure gradient (MPG) and PPG were measured. Thereafter, central plication of both cusps was performed with 5-0 Prolene sutures (Ethicon) until PPG exceeded 20 mm Hg. Finally, the FML was reduced to 26 mm. Then, 0-Ethibond sutures (Ethicon) were passed through both ends of the graft to bridge the 2 commissures of the BAV model and passed through a tourniquet. Six pairs of similar BAV models were created, and the control group was set before the tourniquet was tightened.

### In Vitro Pulsatile Flow Simulator

For the pulsatile simulator, we used a circuit similar to that reported in our previous experiment^4^ (Supplemental Figure). The transaortic pressure difference was measured using a pressure transducer (UK-801; Baxter), and transaortic forward and reverse flow were measured using an ultrasonic flow sensor (ME 19 PXN; Transonic) placed 5 cm proximal to the valve. The average of 6 cycles of consecutive data was used as the measured value.

The basic conditions of the simulation circuit were set to a forward flow of 5.0 L/min, pulse rate of 70 bpm, systolic phase ratio of 35%, and aortic pressure was kept at 120/80 mm Hg. The AVA was measured by direct echography (iU22; Philips) from outside of the graft.

After measurement of the control group, the tourniquet was gradually tightened to 2 mm, 4 mm, 6 mm, and 8 mm, to achieve ICD 24 mm (ICD_24_), 22 mm (ICD_22_), 20 mm (ICD_20_), and 18 mm (ICD_18_), respectively, and the same measurements were made. This was done 3 times for 6 BAV models, and the average of the total of 18 measurements was calculated.

## Results

**Figure 3:**
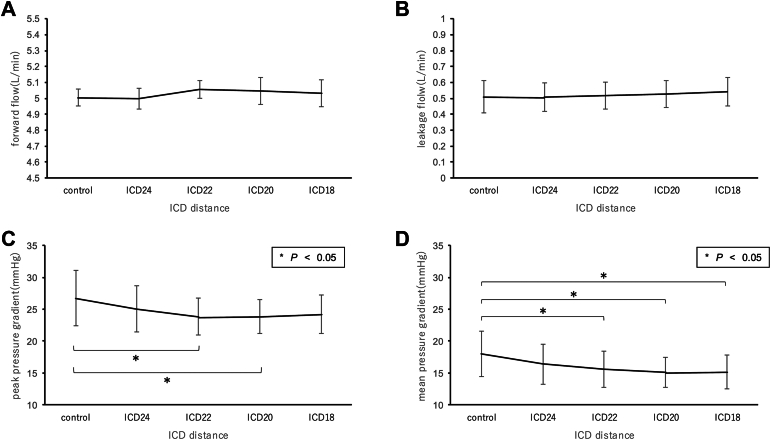
Hydrodynamic results according to the various intercommissural distances (ICDs): (A) Forward flow; (B) Leakage; (C) Peak pressure gradient; (D) Mean pressure gradient.

The results are shown in Figure 3. Forward flow remained constant at around 5.0 L/min. Leakage increased slightly with ICD shortening, but the effect was not significant (control 0.47 ± 0.10 L/min vs ICD_18_ 0.52 ± 0.09 L/min, *P* = .17). PPG and MPG were significantly reduced by ICD shortening (PPG: control 26.75 ± 4.33 mm Hg vs ICD_22_ 23.85 ± 2.91 mm Hg, *P* < .05; MPG: control 17.57 ± 3.59 mm Hg vs ICD_20_ 14.76 ± 2.40 mm Hg, *P* = .01). However, while there were marked decreases up to ICD_24_ and ICD_22_, the pressure gradient remained almost unchanged with further shortening. At ICD_18_, the pressure gradient became slightly larger than at ICD_20_.

**Figure 4:**
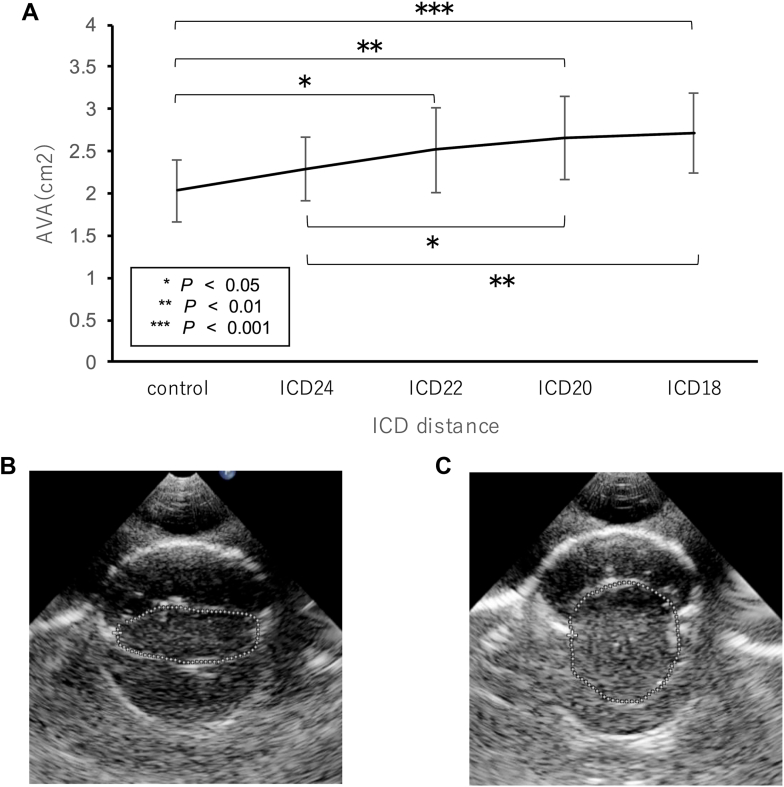
Aortic valve area (AVA) and echocardiographic finding: (A) Aortic valve area according to the various intercommissural distances (ICDs). (B, C) Short-axis view of the aortic valve with ICD of 26 mm (B) or 18 mm (C) during end-systole.

AVA increased significantly with ICD shortening (control 2.03 ± 0.37 cm^2^ vs ICD_18_ 2.71 ± 0.47 cm^2^, *P* < .01) (Figure 4). Although the changes were still moderate at ICD_20_ and ICD_18_, the AVA increased constantly up to ICD_18_.

Marked improvement in valve opening was seen after ICD shortening (Supplemental Video 2: ICD = 18 mm) compared with control (Supplemental Video 1: ICD = 26 mm).

## Comment

The current results clearly showed that shortening the ICD significantly reduced the MPG and PPG and significantly increased the AVA.

Aortic valvuloplasty has been reevaluated in recent years since Aicher and colleagues^1^ reported that BAV repair towards 180° symmetry can be performed with good results. However, a new concern has emerged in asymmetric BAV repair: If cusp plication is too tight, the AVA is reduced, resulting in aortic stenosis. A postoperative high PPG is considered to be associated with a high risk of reoperation,^2^ and very asymmetrical BAV is recommended to be repaired by tricuspidization because of the high risk of aortic stenosis if repaired as a 180° symmetric BAV.^3^ However, tricuspidization is highly technical-demanding, and there is a risk of recurrence due to thickening and shortening of the pericardium. In such cases, symmetric repair must be considered, and countermeasures to avoid stenosis must be taken. Although there have been many studies with computer simulations, they have not been able to correctly reproduce the hemodynamics in clinical practice. Therefore, we conducted hydrodynamic studies with a pulsating simulation circuit, as in the previous studies.^4^^,^^5^

In this study, improvements in pressure gradient and AVA were significant only in ICD_24_ and ICD_22_. Furthermore, the final PPG was never less than 20 mmHg. We initially planned to suture the entire circumference to shrink the entire sinotubular junction (STJ), but the effectiveness of sinus plication, which involves suturing the area from the Valsalva sinus to the STJ, has already been recognized.^4^ Therefore, in order to clarify that shortening the ICD improves the opening, we decided to shorten only the interconnection in this experiment. In this technique, shortening of the commissure position alone does not relieve tension near the annulus, and suture contraction causes strain with a snowman-like shape on the graft; this may be why there was only a slight improvement in pressure gradient between ICD_20_ and ICD_18_. For acceptable durability of BAV repair, the entire aortic root complex should be addressed. Shaping the STJ into its ideal morphology (eg, elliptical shape) or interventions including the aortic root complex, such as sinus plication, may be more effective.^3^^,^^6^

Our final goal is to invent elliptical STJ ring to facilitate BAV repair. Recently, double annuloplasty has been advocated to improve durability of aortic valvuloplasty.^7^^,^^8^ Alternatively, even if only single annuloplasty is performed for BAV repair, this method could be expected as a quick and rescue technique if significant stenosis is observed after aortic unclamping. This study may provide evidence for the importance of ICD shortening and the ideal standard values of ICD. Indeed, other fluid-structure interaction simulations identified that no stenosis occurred when FML was 1.5 times or more the annular diameter, which seems similar to the current findings.^9^

### Limitations

This experimental study had several limitations. First, with an experimental model, it is difficult to reproduce the mobility and elasticity of actual human leaflets and aortic tissue. Although no worsening of regurgitation was observed, shortening the commissure usually carries a risk of valve prolapse. However, in a fluid-structure interaction simulation model, no regurgitation was observed even when FML was 1.5 times or more the annular diameter.^9^ Further research is needed on effective height and coaptation height. Furthermore, tap water was used and the viscosity of blood was not taken into consideration. Even with these drawbacks, our experimental model seems to be reliable due to the reasonable findings.

Second, this study is only basic research and there are many issues regarding its clinical application. As the suture passing through the commissure is directly exposed to the blood flow, turbulence and thrombus adhesion should be evaluated before clinical use. However, in mitral valve repair, the artificial chordae using expanded polytetrafluoroethylene have been widely used without clinically relevant sequelae. Furthermore, a concept similar to ours is being applied to percutaneous mitral valve repair to reduce septal-to-lateral mitral annular distance as a “bridge technique.”^10^ However, as we have mentioned before, our final goal is to develop an elliptical STJ ring, so these concerns will be resolved in the future.

Third, echocardiographic evaluation of AVA is prone to bias due to large differences between cross-sections. We minimized this bias by limiting the measurement to 1 investigator (SH).

### Conclusions

Our experiments using a pulsatile simulation circuit confirmed that shortening of the ICD significantly reduced the PPG and MPG and also significantly increased the AVA. When performing symmetrical BAV repair, it is considered effective to sufficiently shorten the ICD (= STJ diameter) to reduce the tension on the cusps when the risk of stenosis is high.

## Figures and Tables

**Supplementary Figure 1:**
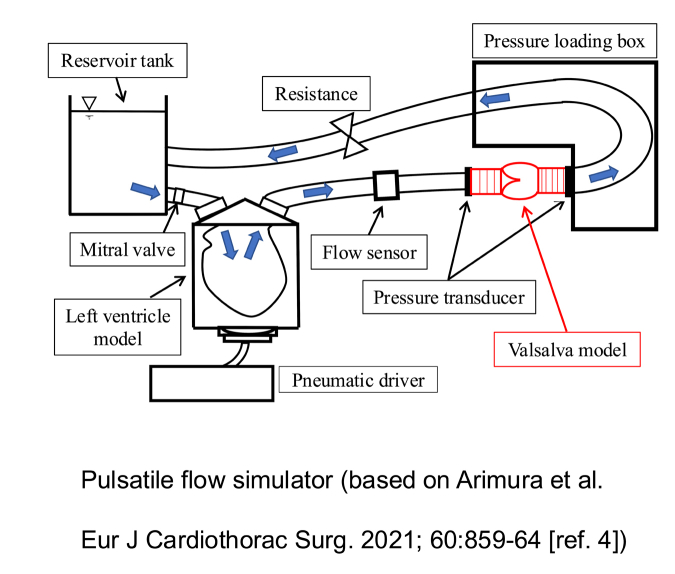


## References

[1] Aicher D., Kunihara T., Abou Issa O., Brittner B., Gräber S., Schäfers H.J. (2011). Valve configuration determines long-term results after repair of the bicuspid aortic valve. Circulation.

[2] Vohra H.A., Whistance R.N., de Kerchove L., Glineur D., Noirhomme P., El Khoury G. (2013). Influence of higher valve gradient on long-term outcome after aortic valve repair. Ann Cardiothorac Surg.

[3] Michelena H.I., Corte A.D., Evangelista A. (2021). International consensus statement on nomenclature and classification of the congenital bicuspid aortic valve and its aortopathy, for clinical, surgical, interventional and research purposes. Eur J Cardiothorac Surg.

[4] Arimura S., Takada J., Nishimura G. (2021). The efficacy of sinus plication in aortic valvuloplasty for bicuspid aortic valve: experiments in a pulsatile flow simulation model. Eur J Cardiothorac Surg.

[5] Kasegawa H., Iwasaki K., Kusunose S. (2012). Assessment of a novel stentless mitral valve using a pulsatile mitral valve simulator. J Heart Valve Dis.

[6] Schneider U., Schmied W., Aicher D., Giebels C., Winter L., Schäfers H.J. (2017). Sinus plication to improve valve configuration in bicuspid aortic valve repair-early results. Ann Thorac Surg.

[7] Shraer N., Youssefi P., Zacek P. (2024). Bicuspid valve repair outcomes are improved with reduction and stabilization of sinotubular junction and annulus with external annuloplasty. J Thorac Cardiovasc Surg.

[8] Benhassen L.L., Hedensted J.H., Sharghbin M. (2023). Comparison of aortic valve repair techniques with single- and double-ring annuloplasties. Eur J Cardiothorac Surg.

[9] Kaiser A.D., Haidar M.A., Choi P.S., Sharir A., Marsden A.L., Ma M.R. (2024). Simulation-based design of bicuspidization of the aortic valve. J Thorac Cardiovasc Surg.

[10] Tozzi P., Locca D., Siniscalchi G., Ait-Tigrine S. (2022). Percutaneous reduction of septal-to-lateral mitral annular distance to increase mitral leaflet coaptation length: preclinical study results. JTCVS Tech.

